# St. Mary's Hospital

**Published:** 1893-07-08

**Authors:** 


### ST. MARY'S HOSPITAL, PADDINGTON.

A festival dinner in aid of the Clarence Memorial Wing of this hospital took place at the Hotel Metropole on Saturday, July 1st, the Duke of Cambridge presiding. Amongst others present were: Canon Duckworth, Cardinal Vaughan (wearing his cardinal's robes of scarlet and purple). Sir J. Crichton Browne, Colonel Leigh Hunt, Colonel S. Bird, the Hon. H. L. Noel, Colonel FitzGeorge, Dr. Broadbent, Mr. W. Austin Leigh, Mr. George P. Field, Dean of the Medical School, and Mr. T. Ryan, the Secretary. The usual loyal toasts having been proposed and responded to, "The Health of the Duke of York," and presently the toast of the evening "Prosperity to St. Mary's Hospital," were proposed by the Chairman, who said that he intended to follow the advice of Sir J. Crichton Browne, and issue the order to "present alms." Lord Randolph Churchill made an urgent appeal last year, and since then the hospital was the richer for a handsome legacy, but in the present year no substantial legacies has been received, and it was again imperative that the needs of St. Mary's Hospital should be brought prominently before the public. There were difficulties to face. The whole income for the past year had only amounted to £12,900, and the expenditure reached a sum of £20,000. More help was urgently needed for the new wing, which would be an immense acquisition to the hospital, and for which £11,000 had already been obtained. The Duke of Cambridge said he felt it would be said that he was always asking for money, but there was no good end to be served by beating about the bush, and on the present occasion to ask for money was simply his plain duty. He would remind those present that the hospital had been founded in 1845, and although by the original scheme 380 beds were to be provided, there had never been more than 200. His Royal Highness trusted the present appeal would be liberally and generously responded to. Speeches followed by Canon Duckworth, Colonel Stanley Bird, Dr. Broadbent, Mr. Field, and Mr. Austin Leigh.

